# The search for novel treatment strategies for *Streptococcus pneumoniae* infections

**DOI:** 10.1093/femsre/fuaa072

**Published:** 2021-01-05

**Authors:** F Cools, P Delputte, P Cos

**Affiliations:** Laboratory for Microbiology, Parasitology and Hygiene (LMPH), University of Antwerp, Universiteitsplein 1, 2610 Wilrijk, Belgium; Laboratory for Microbiology, Parasitology and Hygiene (LMPH), University of Antwerp, Universiteitsplein 1, 2610 Wilrijk, Belgium; Laboratory for Microbiology, Parasitology and Hygiene (LMPH), University of Antwerp, Universiteitsplein 1, 2610 Wilrijk, Belgium

**Keywords:** *Streptococcus pneumoniae*, virulence, drug development, novel drug targets, immunotherapy, antibiotics

## Abstract

This review provides an overview of the most important novel treatment strategies against *Streptococcus pneumoniae* infections published over the past 10 years. The pneumococcus causes the majority of community-acquired bacterial pneumonia cases, and it is one of the prime pathogens in bacterial meningitis. Over the last 10 years, extensive research has been conducted to prevent severe pneumococcal infections, with a major focus on (i) boosting the host immune system and (ii) discovering novel antibacterials. Boosting the immune system can be done in two ways, either by actively modulating host immunity, mostly through administration of selective antibodies, or by interfering with pneumococcal virulence factors, thereby supporting the host immune system to effectively overcome an infection. While several of such experimental therapies are promising, few have evolved to clinical trials. The discovery of novel antibacterials is hampered by the high research and development costs versus the relatively low revenues for the pharmaceutical industry. Nevertheless, novel enzymatic assays and target-based drug design, allow the identification of targets and the development of novel molecules to effectively treat this life-threatening pathogen.

## Teaser

This review discusses recent progress in anti-pneumococcal treatment strategies, with a focus on novel drug targets.

## Key Points

1. Analogues of already marketed antibiotics are in development to overcome increasing pneumococcal resistance. Several of these are currently undergoing clinical trials.
2. Antibacterial targets, consisting of pneumococcal enzymes essential for viability and survival, are being identified. Target-specific rational drug design, including high-throughput screening (HTS) against a major enzyme followed by elucidation of the structure-activity relationship (SAR) and subsequent lead optimization, shows promise in creating novel antibacterials. Contrarily, discovery of natural antibacterials is being hampered by the difficult growth conditions for microbes producing antibacterials. Furthermore, due to potentially low revenues the pharmaceutical sector is showing less interest in the development of novel antibacterials. Therefore, alternative strategies including modulating the host immune system or inhibiting pneumococcal virulence are gaining scientific attention. However, none of these therapies have currently evolved to clinical trials.

## INTRODUCTION

*Streptococcus pneumoniae*, also called the pneumococcus, is a major human pathogen. It is the leading cause of community-acquired bacterial pneumonia (CABP) and can cause otitis media (OM) and meningitis in children, the elderly and immunocompromised patients (Lundbo and Benfield 2017; Peyrani *et al*. 2019). In the USA, pneumonia was the eight leading cause of death in 2015 (Jindal *et al*. 2015). While incidence rates of CABP vary worldwide, overall incidences between 20 and 100 per 10 000 person-years have been observed, with outliers up to 164.3 per 10 000 person-years in patients older than 80 in the USA and up to 294 per 10 000 person-years in Latin-America (Ferreira-Coimbra, Sarda and Rello 2020). For acute OM, 300 million cases are estimated to be caused by *S. pneumoniae* every year (Monasta *et al*. 2012; Bergenfelz and Hakansson 2017).

In the USA, current therapy for pneumococcal infections in infants and children typically consists of amoxicillin or, in case of non-IgE-mediated allergy, a cephalosporin. Alternatively, levofloxacin, linezolid, clindamycin or vancomycin can be used (Bradley *et al*. 2011). Treatment of CABP in adults is generally done with amoxicillin, doxycycline or a macrolide. In case of comorbidities, a fluoroquinolone or combination therapies such as amoxicillin and a cephalosporin or a macrolide and doxycycline are advised by the America Thoracic Society and Infectious Diseases Society of America (Metlay *et al*. 2019). A similar therapy schedule is proposed for patients with pneumococcal meningitis (van de Beek *et al*. 2016). In Europe, amoxicillin or a tetracycline is primarily advised in patients with lower respiratory tract infections, while macrolides can only be used in countries with low resistance rates. Alternatively, levofloxacin or moxifloxacin may be considered for general use, while cephalosporins are reserved for hospital use only (Woodhead *et al*. 2011; Wiersinga *et al*. 2018). While therapy is in general successful, antibiotic resistance is increasingly observed. According to the United States Centre for Disease Control (CDC), resistance to one or more antibiotics is observed in 30% of pneumococcal infections, and it is estimated that 1.2 million infections per year are caused by resistant pneumococcal strains (Centre for Disease Control (CDC) 2013; Cherazard *et al*. 2017). Similarly in Europe, 10% of invasive *S. pneumoniae* isolates reported in 2008 were not susceptible to penicillin, and large regional differences in pneumococcus prevalence have been observed, from less than 5% in Northern Europe to over 40% in some Southern European countries (Woodhead *et al*. 2011). In 2018, these numbers were confirmed. Resistance occurred in 2.5%–32.3% of all cases in Europe for treatment with macrolides (European Centre for Disease Prevention and Control (ECDC) 2019). Also recently, country-wide resistance rates over 25% have been observed in the USA for macrolides. In Europe, variation is higher, ranging from less than 10% in the northern parts to over 25% in parts of Eastern and Southern Europe (Peyrani *et al*. 2019). So far, resistance to fluoroquinolones remains low in the USA and European Union (Woodhead *et al*. 2011; Kim *et al*. 2016).

Pneumococci asymptomatically colonize the nasopharynx, from where they can migrate to other parts of the airway, thereby generating inflammatory responses and disease. They possess a variety of virulence factors, both bound to the cell wall and excreted, that modulate their virulence. A general overview on the importance of virulence factors is described in detail elsewhere and will not be discussed here (Kadioglu *et al*. 2008; Brooks and Mias 2018).

The polysaccharide (PS) capsule is considered the most important pneumococcal virulence factor, as it is part of the first recognition by the immune system. This capsule is known to be diverse, giving rise to over 90 different pneumococcal serotypes. Current vaccines consist of PS fragments of a selection of these serotypes to induce an immune response (Kim, Seon and Rhee 2017). The first PS capsule vaccine to be licensed was a 14-valent PS vaccine in 1977. This vaccine was quickly expanded to include 23 serotypes, and it is still in use today (Briles *et al*. 2019). Unfortunately, immunogenicity of this vaccine is rather poor (Westerink, Schroeder and Nahm 2012). To overcome this issue, the heptavalent pneumococcal conjugate vaccine (PCV7) was the first conjugate vaccine to be licensed in the USA in 2000. Since then, it has been followed by a 13-valent conjugate vaccine (Briles *et al*. 2019). Currently, a 15-valent and a 20-valent conjugate vaccine are in development (Lee *et al*. 2019; Hurley *et al*. 2020). The introduction of conjugate vaccines dramatically reduced the rates of pneumococcal meningitis through direct and indirect (herd) protection (van de Beek *et al*. 2016; Kwambana-Adams *et al*. 2020). However, replacement of vaccine serotypes by non-vaccine serotypes is occurring, thereby lowering vaccine effectiveness. While in the USA this effect is limited, in the UK non-PCV13 serotypes were responsible for over 40% of invasive pneumococcal diseases in 2017 (Deng *et al*. 2013; Kwambana-Adams *et al*. 2020). Still, global vaccination programs are considered essential in the battle against pneumococcal diseases (World Health Organisation (WHO) 2019; Kwambana-Adams *et al*. 2020).

A recent review by Koulenti *et al*. describes in depth all antibacterial agents against Gram-positive bacteria currently in clinical trials (phase I to phase III) (Koulenti *et al*. 2020). Table 1 lists all evaluated compound libraries based on in-use antibiotics since 2010. Such an approach leads to a better understanding of the structure-activity relationship (SAR) of current antibiotics and can therefore lead to the identification of a novel antibiotic-analogue. As such, Table 2 lists a comprehensive overview of the more extensively studied novel antibiotic-analogues since 2010. Their mechanism of action is similar to that of an already marketed antibiotic, but in most cases resistance mechanisms differ and/or increased activity towards resistant strains is observed. Several of these analogues are currently undergoing clinical trials.

**Table 1.** Overview of known antibiotics for which derivatives have been constructed and evaluated against *S. pneumoniae* since 2010.

Library derived from	Antibiotic class	Number of tested molecules in library	References
Vancomycin	Glycopeptides	22	(Chang *et al*. 2013)
		31	(Shao *et al*. 2011)
Lincomycin	Lincosamides	14	(Kumura *et al*. 2016)
		13	(Umemura *et al*.^2013^)
Azithromycin	Macrolides	23	(Fajdetić *et al*. 2011)
		17	(Pavlović and Mutak 2016 )
		30	(Ma *et al*. 2011b)
		28	(Li *et al*. 2013)
		36	(Wang *et al*.2017b )
		8	(Wang *et al*.2017a )
		13	(Čipčić Paljetak *et al*. 2016)
Clarithromycin	Macrolides	10	(Čipčić Paljetak *et al*.^2016^)
		18	(Jia *et al*. 2018)
		24	(Jia *et al*. 2017)
		26	(Qin *et al*.^2018^)
		14	(Liang *et al*. 2012)
		67	(Kumar *et al*. 2012)
		18	(Cong *et al*. 2011)
		33	(Ma *et al*. 2011a)
Erythromycin A	Macrolides	11	(Qi *et al*. 2010)
		8	(Zheng *et al*. 2016)
		26	(Sugimoto *et al*. 2012)
		11	(Bukvić Krajačić *et al*. 2011)
Ketolide	Macrolides	10	(Pereira and Fernandes 2011)
		3	(Chen *et al*. 2012)
		36	(Ma *et al*. 2019)

**Table 2.** Overview of novel antibiotic-analogues against *S. pneumoniae* discovered since 2010. NDA: New drug application; FDA: US Food and Drug Administration.

Name	Year of discovery of anti-pneumococcal activity	Antibiotic class	Clinical trials	References
**Avarofloxacin (JNJ-Q2, acorafloxacin)**	2010	Fluoroquinolones	Phase I	(Morrow *et al*. 2010; Fernandez *et al*. 2011; Covington *et al*. 2013)
**Solithromycin (CEM-101)**	2010	Ketolides (Macrolides)	Phase III	(McGhee *et al*. 2010; Rodgers, Frazier and Champney 2013; Farrell, Mendes and Jones 2015; Zhanel *et al*. 2016; Kato *et al*. 2019)
**MX-2401**	2011	Lipopeptides	None	(Dugourd *et al*. 2011; Rubinchik *et al*. 2011)
**Cefilavancin (TD-1792)**	2012	Glycopeptide-cephalosporin conjugate	Phase III	(Hegde *et al*. 2012)
**Lefamulin**	2013	Pleuromutilins	FDA approved	(Ross *et al*. 2012; Paukner *et al*. 2013; Mendes *et al*. 2016; Paukner and Riedl 2017)
**Contezolid (MRX-1)**	2014	Oxazolidinones	Phase III	(Shinabarger 1999; Li *et al*. 2014)
**Omadacycline (PTK 0796)**	2014	Tetracyclines	FDA approved	(Draper *et al*. 2014; Macone *et al*. 2014)
**RBx 14 255**	2014	Ketolides (Macrolides)	Preclinical	(Raj *et al*. 2014; Barman *et al*. 2019)
**Eravacycline (TP-434)**	2015	Tetracyclines	NDA filed	(Grossman *et al*. 2012, 2015)
**Lascufloxacin**	2017	Fluoroquinolones	NDA filed	(Kishii, Yamaguchi and Takei 2017)
**Nafithromycin (WCK 4873)**	2017	Ketolides (Macrolides)	Phase II	(Zhanel *et al*. 2002; Flamm, Rhomberg and Sader 2017)
**TP-271**	2017	Fluorocyclines (Tetracyclines)	Phase I	(Grossman *et al*. 2017)
**KBP-7072**	2019	Tetracyclines	Phase I	(Lepak *et al*. 2019)

While evaluating derivatives of known antibiotics can be useful, evaluation of new antibacterial targets may also help to overcome resistance mechanisms. As such, targeting bacterial virulence instead of bacterial physiology should be considered (Rasko and Sperandio 2010). Identifying these novel targets is often done using computational screening, in which bacterial and human genome sequences are screened to identify essential bacterial proteins, without affecting the human host and/or its microbiome (Wadood *et al*. 2018; Nayak *et al*. 2019).

Clearly, with antibiotic resistance still increasing worldwide, the search for novel antibacterials remains of utmost importance with several ingoing discovery and development programs. In this review, we will provide a comprehensive overview of the current pneumococcal drug pipeline, hereby excluding known antibiotic analogues and focusing on the discovery of novel drug targets. Overall, novel therapies can be divided into three main categories: (i) modulating the host immune system to lower pneumococcal disease, (ii) interference with pneumococcal virulence and (iii) development of novel antibiotics.

### Modulating the host immune system

**Figure 1.**
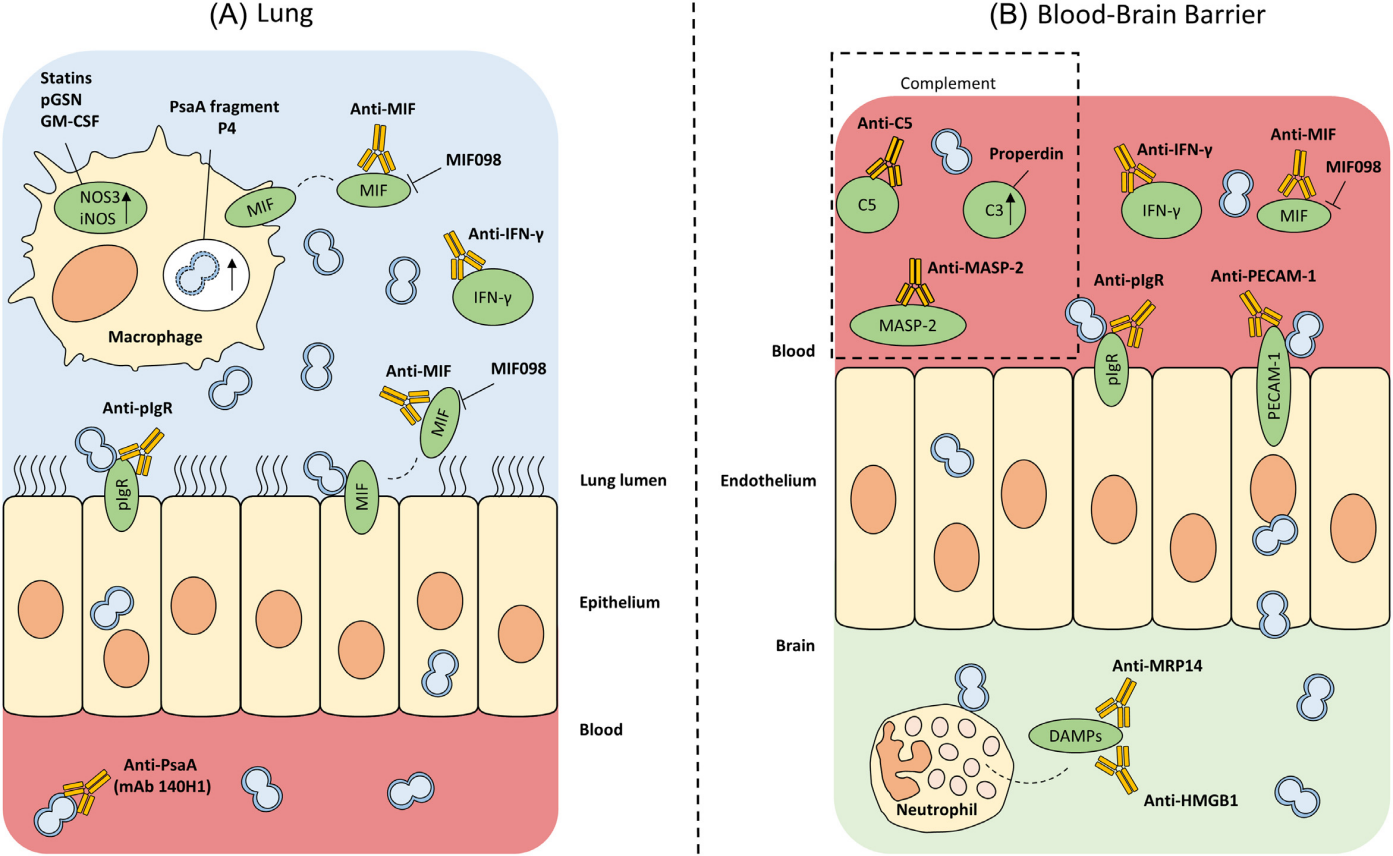
Overview of drug targets and novel therapies focusing on modulating the host immune system at different locations of the body. **(A)**, Drug targets present at air-blood interface. **(B)**, Drug targets present at blood-brain barrier. Drug targets in and on macrophages, on epithelial and endothelial cells, in the blood and in the brain are labeled in green. pGSN: plasma gelsolin, GM-CSF: granulocyte/macrophage-colony stimulating factor, iNOS: inducible nitric oxide synthase, NOS3: nitric oxide synthase-3, PsaA: pneumococcal surface antigen A, MIF: macrophage inhibitory factor, IFN-γ: interferon-γ, pIgR: polymeric immunoglobulin receptor, mAb: monoclonal antibody, MASP-2: mannose-binding lectin-associated serine protease, PECAM-1: platelet endothelial cell adhesion molecule.

Several recently investigated anti-pneumococcal drug targets focus on enhancing host immune responses after infection. Interfering with these responses is challenging, as this might provoke an unwanted immune cascade leading to an increase in inflammatory damage on the one hand or it might overly inhibit the immune system leading to an uncontrolled growth and invasion of pathogens. Figure 1 shows a schematic overview of all discussed therapies. Detailed results regarding the *in vivo* data of these therapies are listed in Table 3.

**Table 3.** *In vivo* results of therapies modulating the host immune system. p.i.: post-infection.

Compound	*In vivo* model	Treatment schedule	Endpoint	References
**Pravastatin**	Murine post-influenza secondary pneumonia model	100 mg/kg intraperitoneally, starting one day before pneumococcal challenge, repeated once daily	Increase in murine survival from 0% to 50% 13 days p.i.	(Yang *et al*. 2014b)
**pGSN**	Murine post-influenza secondary pneumonia model	400 mg/kg subcutaneously, starting one day before pneumococcal challenge, repeated once daily	Reduction in bacterial burden, 50% reduced acute inflammation 24 h p.i.	(Yang *et al*. 2015)
**pGSN**	Murine pneumonia model	5–10 mg/mice intraperitoneally, 2 and 3 days p.i.	Increase in murine survival from 15% to 50% 10 days p.i.	(Yang *et al*. 2019b)
**pGSN + penicillin**	Murine pneumonia model	5–10 mg/mice intraperitoneally + 0.1–2 mg penicillin intramuscularly, starting one day p.i., repeated once daily	Increase in murine survival from 30% to 80% 10 days p.i., decrease in weight loss and overall morbidity score	(Yang *et al*. 2019b)
**GM-CSF**	Murine pneumonia model	20 µg/mouse orotracheally, 6 h p.i.	1-log reduction in bacterial burden, 2-fold increase in macrophages present in murine lung exudate 24 h p.i.	(Steinwede *et al*. 2011)
**MIF antibodies**	Lethal murine sepsis model	2 mg/mouse intraperitoneally, 2 h prior to infection	Increase in murine survival from 25% to 53.6% 15 days p.i., almost 4-log reduction in bacterial burden 48 h p.i.	(Savva *et al*. 2016)
**MIF098**	Murine pneumonia model	40 mg/kg intraperitoneally, twice daily	Increase in murine survival from 10% to 50% 7 days p.i., 2-log reduction in bacterial burden 48 h p.i., reduced neutrophil and monocyte infiltration 48 h p.i.	(Weiser *et al*. 2015)
**anti-pIGR and anti-PECAM-1 antibodies**	Murine meningitis model	4 µg/mouse intravenously, 1 or 5 h p.i.	Prolonged survival of mice, 1-log reduction in bacterial burden after succumbing	(Iovino, Thorsdottir and Henriques-Normark 2018)
**anti-pIGR and anti-PECAM-1 antibodies + ceftriaxone**	Murine meningitis model	4 µg/mouse antibodies + 100 mg/kg ceftriaxone intravenously, 1 h p.i.	Increase in murine survival from 60% to 100%, reduction in bacterial burden, prevention from passing the BBB in 60% of all cases, reduced neuroinflammation 5 days p.i.	(Iovino, Thorsdottir and Henriques-Normark 2018)
**C5 antibodies**	Murine meningitis model	1 mg/mouse intraperitoneally, 24 h p.i.	Increase in murine survival from 66% to 100%, visible reduction in cerebral hemorrhages 48 h p.i.	(Woehrl *et al*. 2011)
**C5 antibodies**	Murine meningitis model	1 mg/mouse intraperitoneally, 20 h p.i.	Increase in murine survival from 10% to 30% 72 h p.i.	(Kasanmoentalib *et al*. 2015)
**MASP-2 antibodies**	Murine meningitis model	1 mg/kg intraperitoneally, 20 h p.i.	Increase in murine survival from 64% to 86%, brain burden unaffected 68 h p.i.	(Kasanmoentalib *et al*. 2017)
**Recombinant FH**	Murine sepsis model	600 µg/mouse intraperitoneally + 25 mg/kg ceftriaxone, 17 h p.i.	No effect of treatment on disease score, cytokine production or vascular leakage in the liver 26 h p.i.	(Van Der Maten *et al*. 2016)
**Recombinant FH**	Murine meningitis model	1 mg/mouse intraperitoneally, 16 h p.i.	No effect of treatment on murine survival 72 h p.i. or bacterial burden 24 h p.i.	(Kasanmoentalib *et al*. 2019)
**Properdin**	Murine intranasal infection model	100 µg/mouse intraperitoneally, at time of infection	Increase in murine survival from 0% to 90% 60 h p.i., 2-log reduction in blood burden 24 h p.i.	(Ali *et al*. 2014)
**IFN-γ antibodies**	Murine meningitis model	30 µg/mouse intracranially, at time of infection	Increase in murine survival from 33% to 83% 4 days p.i., brain burden unaffected 48 h p.i.	(Pettini *et al*. 2015)
**Anti-DR5 antibodies**	Murine pneumonia model	75 µg/mouse intratracheally, 6 h p.i.	Increase in murine survival from 30% to 70% 8 days p.i., 1-log reduction in bacterial lung burden 72 h p.i.	(Steinwede *et al*. 2012)
**Paquinimod**	Murine meningitis model	10 mg/kg intracranially, 24 h p.i.	Reduction of cranial inflammation with over 50% reduction in white blood cell count in the brain and CXCL2 levels 48 h p.i.	(Wache *et al*. 2015)
**HMGB1 antibodies**	Murine meningitis model	100 µg/mouse intraperitoneally, 21 h p.i.	Increase in murine survival from 25% to 100%, improved clinical parameters (e.g. temperature), 58% reduction in white blood cell count in the brain 45 h p.i.	(Masouris *et al*. 2017)
**Peptide P4**	Murine intranasal infection model	100 µg/mouse intravenously, 48 h and 72 h p.i.	Increase in murine survival from 45% to 95% in 11-month-old mice, from 20% to 73% in 15-month old mice and from 30% to 80% in 6 to 10 weeks old mice 144 h p.i.	(Rajam *et al*. 2010)
**mAB 140H1 (PsaA antibody)**	Murine pneumonia model	200 µg/mouse intraperitoneally, 6 h p.i.	Increase in murine survival from 0% to 54% 15 days p.i., 2-log reduction in lung burden and full clearance in blood 24 h p.i.	(Kristian *et al*. 2016)
**mAB 140H1 (PsaA antibody) + ceftriaxone**	Lethal murine sepsis model	100 µg/mouse antibody + 50 mg/kg ceftriaxone intraperitoneally, 24 h p.i.	Increase in murine survival from 50% to 100% 15 days p.i.	(Kristian *et al*. 2016)

### Induction of nitric oxide synthase (NOS) activity

After macrophage activation by a variety of pro-inflammatory cytokines, constitutively expressed nitric oxide synthase-3 (NOS3) and inducible NOS (iNOS) expression are increased. These synthases show bactericidal activity through the production of nitric oxide (NO) (Hernansanz-Agustín *et al*. 2013). NO is used as a signaling molecule in low concentrations, while in high concentrations (e.g. during the oxidative burst in neutrophils) it shows direct antimicrobial properties by binding to DNA, proteins and lipids (Schairer *et al*. 2012). Although an excess production of NO can lead to immunosuppression, NOS inhibitors have previously been reported to reduce tissue injury and mortality in pneumococcal meningitis models. (Fang 2004) It is clear that a strict regulation of the amount of NO is needed to battle an infection. NOS3 expression is mediated by estrogen (Yang *et al*. 2014b, 2015). This estrogen-dependency leads to a greater risk of developing pneumonia for males than females (Casimir *et al*. 2013; Yang *et al*. 2014b). Statins, known to boost NOS3 activity, have been shown to improve bacterial clearance and survival from secondary pneumococcal pneumonia (Yang *et al*. 2014b). Furthermore, the use of statins has proven to be beneficial to patients suffering from pneumonia and is associated with a lower risk of hospitalization and mortality (Nielsen *et al*. 2012; Nishimoto, Rosch and Tuomanen 2020). Administration of sub-cutaneous plasma gelsolin (pGSN), a human blood protein, has also been shown to activate NOS3 and improve outcome of secondary bacterial pneumonia in mice, with reduced acute inflammation and improved bacterial clearance after 24 hours (Yang *et al*. 2015). The positive effects of pGSN treatment were confirmed in a more clinically relevant murine treatment model using an antibiotic-resistant pneumococcal strain (Yang *et al*. 2019b). Furthermore, in a 2019 study, patients suffering from community-acquired pneumonia (CAP) admitted to the hospital with low pGSN concentrations were more at risk for developing severe, short-term clinical outcomes such as higher risk of death, septic shock and respiratory failure (Self *et al*. 2019). Recently, a phase 1 clinical trial was finalized showing recombinant pGSN was generally safe and well tolerated in patients with mild CAP symptoms and a clinical trial studying the use of recombinant pGSN for the treatment of Covid-19 patients is currently recruiting (Tannous *et al*. 2020). Apart from NOS3, increased levels of iNOS are also known to contribute to the antibacterial activity of alveolar macrophages against pneumococci. It has also been shown that granulocyte/macrophage-colony stimulating factor (GM-CSF), used in the treatment of leukemia, is able to induce iNOS induction in response to infection, leading to a reduction in bacterial load and inflammation in the lungs of mice (Steinwede *et al*. 2011).

### Macrophage inhibitory factor (MIF) inhibitors

MIF is a component of the innate immune system and constitutively expressed by immune and epithelial cells. It is released rapidly after exposure to bacteria and pro-inflammatory cytokines, promotes expression of numerous other pro-inflammatory molecules and thereby amplifies the response. Apart from its role in inflammation and immunity, it is also important in cell proliferation and oncogenesis (Roger *et al*. 2007; Bewersdorf *et al*. 2018). While MIF is essential for pneumococcal clearance after nasopharyngeal colonization (Das *et al*. 2014), high MIF levels in cerebrospinal fluid (CSF) are associated with poor patient outcome. Moreover, mice treated with MIF-neutralizing antibodies show lower bacterial burdens in lungs and a higher survival in a lethal pneumococcal sepsis model (Savva *et al*. 2016). Similarly, in another study using a murine lung infection model, treatment with the small-molecule receptor antagonist MIF098 improved survival, decreased bacterial burdens by 2 logs and reduced inflammation (Weiser *et al*. 2015).

### Blocking of brain endothelial receptors

Adhesion to and invasion of endothelial and epithelial cells, mediated by several cellular receptors, is an important aspect of invasive pneumococcal disease, such as meningitis. First, the platelet-activating factor (PAF) receptor on activated epithelial and endothelial cells enables the bacteria to enter the basal membrane of the host epithelial cell. Secondly, the pneumococcal choline-binding protein PspC can bind to the epithelial polymeric immunoglobulin receptor (pIgR). Analogous to the PAF receptor pathway, bacteria are transported into the cell. Concordantly, when crossing the blood-brain barrier (BBB), the PAF receptor is used again (Koedel, Scheld and Pfister 2002; Mook-Kanamori *et al*. 2011). Furthermore, also pIgR is expressed by brain endothelial cells and can be used by pneumococci to adhere. Lastly, platelet endothelial cell adhesion molecule (PECAM-1), one of the major endothelial adhesion molecules, can mediate adhesion of pneumococci to the BBB endothelium (Iovino *et al*. 2017). Using an *in vivo* meningitis model, treatment of infected mice with anti-pIgR and anti-PECAM-1 antibodies 1 hour post infection increased survival time and lowered bacterial brain burden, yet all mice eventually still succumbed. A co-treatment strategy with ceftriaxone was however more successful. Ceftriaxone was capable of clearing the blood infection while the anti-pIgR and anti-PECAM-1 antibodies prevented most bacteria from passing the BBB, leading to a decrease in bacterial burdens and an increase in survival. Moreover, neuroinflammation was significantly lower in the combination therapy group compared to untreated mice or mice treated with ceftriaxone alone (Bewersdorf *et al*. 2018; Iovino, Thorsdottir and Henriques-Normark 2018).

### Modulation of complement activity

The complement system is important in human immunity and comprises several recognition proteins activated in response to pathogens. Activation is triggered by a proteolytic cleavage amplification cascade, which generates fragments capable of binding to microbial surfaces as opsonins and bacterial cell destructions. Opsonins can also promote pathogen phagocytosis and induce inflammatory responses (Andre *et al*. 2017). In pneumococcal meningitis, complement however has a dual role. While it is needed to initiate complement-mediated bacterial killing, uncontrolled activation can occur and often leads to a worse disease outcome (Bewersdorf *et al*. 2018). High levels of complement component 5 (C5) have been shown to worsen patient outcome for bacterial meningitis. Furthermore, adjuvant therapy with C5 antibodies showed beneficial effects on survival rates, brain damage and clinical severity in several murine *in vivo* studies (Woehrl *et al*. 2011; Kasanmoentalib *et al*. 2015). Similar results have been found using mannose-binding lectin-associated serine protease (MASP-2), an important activator in the lectin pathway. While MASP-2 has proven to be important in avoiding nasopharyngeal carriage of pneumococci, it is also associated with worsened meningitis outcomes. As with C5, treatment with MASP-2 antibodies after pneumococcal infection increased *in vivo* murine survival (Kasanmoentalib *et al*. 2017). Complement factor H (FH), a regulatory protein inhibiting complement component 3 (C3), is known to be important in moderating pneumococcal disease. However, in several mouse models, combination therapy of recombinant FH with ceftriaxone showed no beneficial effects (Van Der Maten *et al*. 2016; Kasanmoentalib *et al*. 2019). Lastly and contradictorily to aforementioned therapies, Ali *et al*. showed increased *in vitro* opsonization of pneumococci after properdin treatment, i.e. a known positive regulator of complement activation naturally present in humans. Furthermore, animals infected with pneumococci and treated with properdin show a higher chance of survival and lower bacterial blood burden compared to non-treated animals (Ali *et al*. 2014). Clearly, functions of complement in pneumococcal pathogenesis are diverse. Interfering with its working mechanism might result in major and unforeseen changes in immune responses, and therefore, this interference should be done in a controlled and temporarily manner. Currently, one anti-C5 antibody, eculizumab, is on the market. However, side effects to this drug include increased risk of severe meningococcal meningitis, demonstrating the delicate role of the complement system in bacterial infections. Two other antibodies are currently undergoing clinical trials (Koelman, Brouwer and Van De Beek 2019).

### Interferon-gamma (IFN-γ) inhibitors

Similarly to the aforementioned molecules, IFN-γ also plays a dual role in pneumococcal disease. As a powerful mediator of different innate and adaptive immune pathways during inflammation and infection, it is required for generating an effective response upon bacterial invasion. However, it is also involved in long-term neurological sequelae after pneumococcal meningitis and interfering with IFN-γ responses can lead to a higher survival rate of patients (Too *et al*. 2014; Yau *et al*. 2017; Bewersdorf *et al*. 2018). IFN-γ antibody treatment improved survival after pneumococcal meningitis and overall clinical symptoms were less compared to non-treated animals. Interestingly, bacterial burdens were unaffected by this treatment (Pettini *et al*. 2015).

### Death receptor (DR) agonists

Tumor necrosis factor-related inducing ligand (TRAIL) is a member of the TNF superfamily. There are five human TRAIL receptors known, of which receptors DR4 and DR5 are involved in apoptosis of neutrophils, amongst other effects (Sag *et al*. 2019). It has been shown that TRAIL deficiency leads to increased inflammation following pneumococcal challenge (Hoffmann *et al*. 2007). In a murine pneumonia model, treatment with agonistic anti-DR5 antibodies led to a small but significant decrease in lung burden 72 h p.i. and an increase in survival from 30% to 70% 8 days p.i (Steinwede *et al*. 2012). However, TRAIL is also considered important in protection against viral lung infection, which often precede pneumococcal infections. Therefore, interference with this molecule is considered difficult, making it less favorable as therapeutic target (Braithwaite, Marriott and Lawrie 2018).

### Danger-associated molecular pattern (DAMP) inhibitors

Meningitis is triggered after recognition of pathogen-associated molecular patterns (PAMPs). As a result, neutrophils are recruited and release toxins in order to kill the pathogens present. However, these neutrophil-derived toxins can, in turn, cause stress and damage to host cells. These injured cells subsequently release DAMPs, responsible for tissue damage (Wache *et al*. 2015; Masouris *et al*. 2017). HMGB1 and MRP14 are DAMPs known to be secreted in large amounts in de CSF of meningitis patients and could thus be a therapeutic target. Recently, the MRP14 inhibitor paquinimod that could be used in autoimmune diseases has been shown to reduce inflammation in a pneumococcal meningitis model, further supporting its potential for meningitis therapy (Wache *et al*. 2015; Bewersdorf *et al*. 2018). Similarly, HMGB1 antibodies were also shown to lower inflammation, and improve the outcome of pneumococcal infection (Masouris *et al*. 2017). As for other immunomodulatory therapies, inhibiting DAMPs can only be done in a very tightly controlled and balanced manner to assure controlled inflammation and subsequent elimination of bacteria (Land 2020).

### Pneumococcal surface antigen A (PsaA) and pneumococcal surface protein A (PspA) as targets for therapy

PsaA and PspA are well-known pneumococcal virulence factors and have been studied as potential vaccine candidates. PsaA is an indispensable protein for Mn^2+^ transport, protecting against oxidative stress and for adherence to endothelial cells. PspA is important in evasion of the host immune system by interfering with the complement C3 cascade (Tai 2006). Mutants lacking PsaA are significantly less virulent compared to their parent strains, which could be attributed to growth impairment, reduced adherence capacity or hypersensitivity to oxidative stress (Rajam *et al*. 2008a). PsaA however, seems also essential in the induction of a protective host immune response, since peptide P4, a short amino acid fragment of PsaA, was capable of binding to nasopharyngeal epithelial cells and eliciting an inflammatory response (Rajam *et al*. 2008b). Furthermore, *ex vivo* human alveolar macrophages and neutrophils demonstrated improved bacterial killing after P4 exposure and treatment of intranasal infected mice with P4 was shown to increase survival (Rajam *et al*. 2009, 2010; Bangert *et al*. 2013; Morton *et al*. 2016). No follow-up studies involving P4 have been reported so far. Monoclonal antibodies (mAbs) against PspA have also been studied for therapeutic use. Several mAbs against PspA were produced in mice, and their *in vitro* opsonization capacity to pneumococci was evaluated. One selected mAb (mAb 140H1) was evaluated *in vivo*, and administration after infection with pneumococci reduced bacterial lung and blood burden and improved survival rate. Furthermore, combination therapy with the standard antibiotic ceftriaxone showed a synergistic effect (Kristian *et al*. 2016). Currently, PspA is studied as a vaccine candidate rather than a therapeutic target (Wagner-Muñiz *et al*. 2018; Akbari *et al*. 2019).

### Interfering with pneumococcal virulence

**Figure 2.**
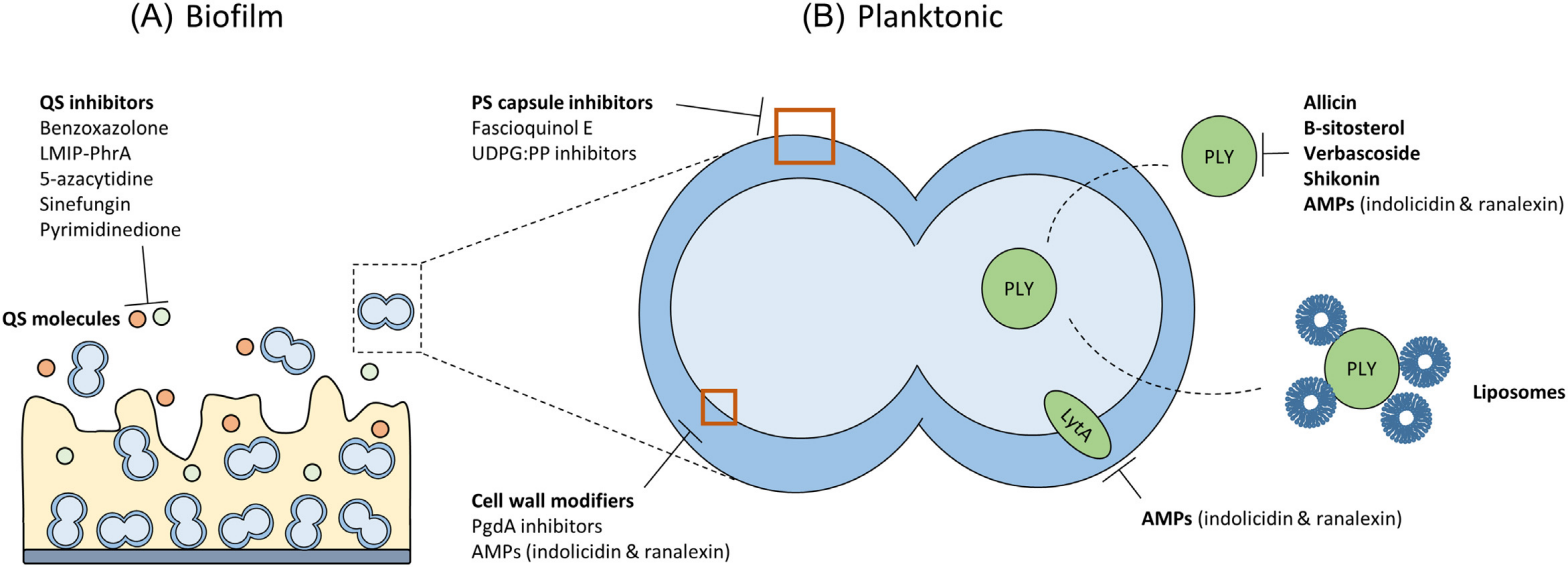
Novel therapies interfering with pneumococcal virulence. **(A)**, Drug targets involved in biofilm formation are targeting quorum-sensing mechanisms. **(B)**, Drug targets present on/in individual pneumococci. Drugs specific for these targets aim at inhibition of polysaccharide capsule, pneumolysin and LytA and modification of the pneumococcal cell wall. QS: quorum sensing, LMIP: linear molecularly imprinted polymer, PS: polysaccharide, UDPG:PP: uridine diphosphate glucose pyrophosphorylase, PgdA: peptidoglycan N-acetylglycosamine deacetylase A, AMPs: antimicrobial peptides, PLY: pneumolysin.

Next to modulating host immunity, research focusing on reducing pneumococcal virulence to enable the immune system to overcome the infection shows growing interest. Most proposed targets are based on virulence factors specific to pneumococci and thus potential novel therapies will be pathogen-specific. A schematic overview of the drug targets is shown in Fig. 2. Table 5 lists detailed results regarding the current *in vivo* data of these therapies.

### PS capsule formation inhibitors

As mentioned earlier, the PS capsule is the most important virulence factor. It inhibits macrophage phagocytosis, which forms the first line of defense to pneumococcal invasion (Dockrell *et al*. 2003; Dockrell, Whyte and Mitchell 2012). Deletion of the capsule increases phagocytosis rates *in vitro* and decreases virulence *in vivo* (Preston and Dockrell 2008). However, downregulation of the capsule is needed to initiate nasopharyngeal colonization (Gilley and Orihuela 2014). Also during pneumonia and OM, downregulation of the capsule and subsequent formation of a biofilm is considered part of the pneumococcal immune evasion strategy (Moscoso, Garcia and Lopez 2006; Domenech *et al*. 2013). In contrast, when the change from a commensal to a pathogenic lifestyle occurs, pneumococci upregulate their PS capsule production (Gilley and Orihuela 2014). The pneumococcal capsule is formed through two pathways, the Wzy-dependent pathway used by most, and the synthase-dependent pathway, only used in serotypes 3 and 37 (Geno *et al*. 2015). The capsule of serotype 3 consists of glucose (Glc) and glucuronic acid (GlcA) and is the result of 3 genes (*cps3D*, *cps3S* and *cps3U*), of which only the first two are essential genes for capsule production. Serotype 37 only requires one gene, *tts*, to form its capsule, consisting solely of Glc chains. The Wzy-dependent pathway is more complex. In general, sugar-1-phosphate is transferred to a lipid carrier on the cytoplasmic side of the cell membrane by a glycosyltransfersase. From there, the repeat unit is built and translocated to the extracellular side by flippase Wzx before being polymerized by Wzy. Lastly, the final sugar (usually Glc) is covalently bound to N-acetylglucosamine residue of the cell wall peptidoglycan (Geno *et al*. 2015; Paton and Trappetti 2019). While these pathways differ, they both use the *cps* gene locus to build the correct sugar conformation. As the loci slightly differ for each serotype, interfering with them or their products is difficult. Thus far, only CpsB, a tyrosine phosphatase encoded by *cpsB*, has been suggested to be a potential novel anti-virulence drug target, as *cpsB* mutants are avirulent in several animal models of infection (Morona *et al*. 2004; Standish *et al*. 2012; Monteiro Pedroso *et al*. 2017). Furthermore, the molecule fascioquinol E—an extract derived from the marine sponge *Fasciospongia spp.—*inhibited CpsB phosphatase activity and increased macrophage attachment *in vitro* (Standish *et al*. 2012).

Lastly, regardless of the pneumococcal serotype or capsule pathway, uridine diphosphate glucose (UDP:Glc) is generally considered a key component in the formation of PS capsule. UDP:Glc is part of the Glc and galactose (Gal) metabolism and is made through the interconversion of glucose-1-phosphate (Glc-1-P) by uridine diphosphate glucose pyrophosphorylase (UDPG:PP) (Mollerach, López and García 1998). It has been described that mutants lacking a functional *galU* gene form a lower amount of PS capsule, are more prone to macrophage phagocytosis *in vitro* and are less virulent in a *Galleria mellonella in vivo* model. (Mollerach, López and García 1998; Cools *et al*. 2018) As the pneumococcal UDPG:PP crystal structure is unknown, modeling of new molecules is challenging. However, recently, two attempts have been made, either using the purified enzyme in an enzymatic assay or using a computational molecular docking model based on other bacterial UDPG:PP's (Zavala *et al*. 2017; Cools *et al*. 2020).

### Bacterial cell wall modifiers

The cell wall of Gram-positive bacteria, such as the pneumococcus, consists of a thick peptidogylycan (PG) layer, to which teichoic acids and capsular PS are covalently attached. Due to its importance, PG biosynthesis has always been an interesting drug target. Currently, beta-lactam antibiotics and vancomycin are the two most used PG inhibitors (Vollmer, Blanot and De Pedro 2008; Rajagopal and Walker 2017; Vollmer, Massidda and Tomasz 2019).

Lysozyme, a major bacteriolytic component of the immune system, hydrolyses PG chains, which results in lysis of bacteria. Pathogens such as *S. pneumoniae*, however, modify their glycan strands through deacetylation by peptidoglycan N-acetylglycosamine deacetylase A (PgdA), thereby increasing resistance to lysozyme. Mutant strains lacking PgdA are significantly more susceptible to lysozyme *in vitro* and show a reduction in virulence *in vivo* (Vollmer and Tomasz 2002). Since then, several publications have described *in vitro* inhibitors of PgdA enzymatic activity, yet no follow-up studies on the activity towards the bacterial cell or *in vivo* use have been performed (Bui *et al*. 2011; Ariyakumaran *et al*. 2015; DiFrancesco, Morrison and Nitz 2018).

### Pneumolysin (PLY) inhibitors

PLY is formed intracellularly and released in the environment through bacterial lysis. It is known to be cytolytic to all human cell types through the formation of pores. Furthermore, it promotes pro-inflammatory immune responses through activation of the classical and lectin pathways of complement activation (Anderson and Feldman 2017). As PLY is known as an essential virulence factor for bacterial survival in the respiratory tract, inhibiting this enzyme might prove beneficial (Kadioglu *et al*. 2008; Kim, Seon and Rhee 2017). A variety of natural compounds has been successfully tested for their anti-PLY activity (Table 4). Also statins were proven to have a direct effect on PLY cytotoxicity *in vitro* and *in vivo* and could be valuable as adjuvant therapy to existing antibiotics. However, further studies are needed to study the mechanism of action and potential place in pneumococcal therapy (Nishimoto, Rosch and Tuomanen 2020).

**Table 4.** Natural compounds tested for their anti-PLY activity since 2010. PLY: pneumolysin.

Natural compound	Source	Activity on PLY	References
**Allicin**	Active component of garlic	Inhibition of hemolytic activity *in vitro*.	(Arzanlou *et al*. 2011)
**β-sitosterol**		Inhibition of hemolytic activity and protection of human lung cells *in vitro*; decrease in mortality, bacterial burden and pulmonary inflammation *in vivo*.	(Li *et al*. 2015)
**Verbascoside**	Glycoside present in plants used in Chinese medicine	Inhibition of hemolytic activity.	(Zhao *et al*. 2016)
**Shikonin**	Component of traditional Chinese herb	Inhibition of hemolytic activity and protection of human alveolar epithelial cells against cell death *in vitro*; reduction of mortality, inflammatory cell infiltration and cell damage in a murine *in vivo* model.	(Zhao *et al*. 2017)
**Juglone**	Roots, leaves, woods and fruits of *Juglandaeae* walnut trees	Inhibition of PLY oligomerization, needed for pore formation.	(Song *et al*. 2017a)
**Epigallocathechin gallate**	Major component of green tea catechins	Inhibition of PLY oligomerization, needed for pore formation; inhibition of sortase A (SrtA) leading to an *in vitro* decrease in biofilm formation; reduction of mortality, lung burden and overall inflammatory reactions a murine *in vivo* model.	(Song *et al*. 2017b)

**Table 5.** *In vivo* results of therapies interfering with pneumococcal virulence. p.i.: post-infection.

Compound	*In vivo* model	Treatment schedule	Endpoint	References
**B-sitosterol**	Murine intranasal infection model	80 mg/kg subcutaneously, 1 h p.i., repeated every 4 h for 48 h	Increase in murine survival from 10% to 70% 120 h p.i., 2-log reduction in bacterial burden 48 h p.i., decrease in pulmonary inflammation 48 h p.i.	(Li *et al*. 2015)
**Verbascoside**	Murine intranasal infection model	100 mg/kg subcutaneously, 2 h p.i.	Increase in murine survival from 25% to 75% 120 h p.i., 1-log reduction in lung burden 48 h p.i., visual pulmonary inflammation is reduced 48 h p.i.	(Zhao *et al*. 2016)
**Shikonin**	Murine intranasal infection model	50 mg/kg orally, 2 h p.i., repeated once daily	Increase in murine survival from 10% to 60% 5 days p.i., 1-log reduction in lung burden, reduction in inflammatory cell infiltration and cell damage 3 days p.i.	(Zhao *et al*. 2017)
**EGCG**	Murine intranasal infection model	50 mg/kg, subcutaneously, directly after infection, repeated in 8 h intervals	Increase in murine survival from 40% to 60% 120 h p.i., 1-log reduction in lung burden 48 h p.i., reduction in overall inflammatory reactions in the lung 48 h p.i.	(Song *et al*. 2017b)
**Liposomes**	Murine intranasal infection model	100 mg/kg intranasally, 30 min p.i.	Increase in murine survival from 40% to 80%, 1-log reduction in lung and blood burden, reduction in inflammatory responses in the lungs 24 h p.i.	(Henry *et al*. 2015)
**Liposomes**	Lethal murine sepsis model	100 mg/kg intravenously, 6 h p.i.	Increase in murine survival from 0% to 50–60%, 4-log reduction in bacterial blood burden, reduction in inflammatory responses in the lungs, 2-fold reduction in blood TNF-alpha levels 24 h p.i.	(Henry *et al*. 2015)
**indolicidin and ranalexin analogues**	Murine pneumonia model	20 mg/kg intraperitoneally, 1 h, 12 h and 24 h p.i.	Increase in murine survival from 0% to 30–50%, clearance of bacteria in blood, reduction in tissue damage in lungs and spleen 7 days p.i.	(Jindal *et al*. 2017)
**indolicidin and ranalexin analogues**	Lethal murine sepsis model	10 mg/kg intraperitoneally, 1 h, 12 h and 24 h p.i.	Increase in murine survival from 0% to 60%, reduction in bacterial burden, decrease in tissue damage in lungs and spleen 7 days p.i.	(Jindal *et al*. 2017)
**Benzoxazolone**	Guinea pig otitis media model	12 mg/kg intraperitoneally, twice daily for 3 months	Prevention of biofilm formation on cochlear implants after 3 months	(Cevizci *et al*. 2015)
**LMIP-PhrA**	Murine intranasal infection model	100 nM/50 µL intranasally, at time of infection	Increase in murine survival from 37 h to 65 h, 2-log reduction in bacterial blood burden 24 h p.i.	(Motib *et al*. 2017)
**Sinefungin**	Rat otitis media model	1,75 µg/rat in the middle ear, at time of infection	0.7 log reduction in burden on bulla 1 week p.i.	(Yadav *et al*. 2014)

Lastly, liposomes consisting of naturally occurring cholesterol and sphingomyelin can be used to sequester PLY (Baumgartner *et al*. 2016). *In vitro*, these liposomes protect monocytes from secreted PLY. Furthermore, mice were more prone to survive a pneumococcal lung infection after treatment with liposomes. Besides a small reduction in bacterial burdens in lung and blood, the treatment was also capable of reducing tumor necrosis factor α (TNF-α) levels. This led to less signs of inflammation in the lungs. In an *in vivo* sepsis model, bacterial blood burden was reduced after treatment, leading to an increase in murine survival. Also in this model, TNF-α levels were decreased (Henry *et al*. 2015). CAL02, a mixture of liposomes, has undergone clinical trials to verify its safety and activity. While a dosage effect could not be established, the safety and tolerability of CAL02 was promising, with no adverse effects that could be linked to local tolerability events. Furthermore, all patients receiving CAL02 treatment were cured 15 to 22 days after the start of the trial (Laterre *et al*. 2019).

### Antimicrobial peptides (AMPs)

AMPs are a large and diverse group of molecules, produced by both pro- and eukaryotes. In humans, they are important in innate immunity, while in bacteria AMPs are produced as a way to compete with and kill other bacteria (Mahlapuu *et al*. 2016). In a 2015 study, analogues of indolicidin and ranalexin, two natural peptides with known antibacterial activity against Gram-positive bacteria, showed *in vitro* bactericidal activity towards antibiotic susceptible and resistant pneumococcal strains, without cytotoxicity for eukaryotic cells (Jindal *et al*. 2015). *In silico* molecular docking pointed towards interactions with autolysin and/or PLY, two known pneumococcal virulence factors as a mechanism of action, while a later study revealed that cell membrane integrity is highly impacted by these AMPs *in vitro* (Jindal *et al*. 2015, 2017). This study also confirmed the *in vivo* effect of these compounds in a murine bacteremia model, leading to an increase in survival, decrease in bacterial burden and decrease in tissue damage in lungs and spleen (Jindal *et al*. 2017).

### Quorum sensing (QS) inhibitors

Biofilm formation is known to play an important role in OM and cochlear implant infections (Cevizci *et al*. 2015). QS systems are known to control the maturation stage of these biofilms, as they allow communication between bacteria in a cell-density dependent manner (Brackman and Coenye 2014). Targeting this system, rather than targeting the bacteria itself, has been gaining scientific attention and *S. pneumoniae* uses several QS systems that have the potential for intervention. First, the ComABCDE pathway is regulated by competence-stimulating peptide (CSP). This system allows for induction of competence and controlling genetic transformation, when a biofilm has matured and pneumococcal density is high enough. Bacteriocins, which inhibit the growth of competing bacteria, are produced as a response to the BlpABCSRF pathway, which operates similarly to ComABCDE. Also, autoinducer-2 (AI-2) is known as a common QS system of Gram-positive bacteria and is also present in pneumococci. The enzyme LuxS activates AI-2, which, in turn, facilitates initial attachment of bacteria to a surface (Galante *et al*. 2014). Apart from LuxS, DNA adenine methyltransferase (DAM) plays an important role in the biosynthesis of AI-2, as it is part of the activated methyl cycle. It catalyzes a methyl transfer from S-adenosyl-L-methionine (SAM) to adenine, a feature unique in bacteria (Yadav *et al*. 2015). Lastly, in 2015, Dimarchi *et al*. discovered a novel QS system, TprA/PhrA, that controls the expression of bacteriocins called lantibiotics (Hoover *et al*. 2015). This system has been shown to be crucial for pneumococcal virulence in pneumonia, meningitis and OM models (Motib *et al*. 2017).

Several biofilm disruption strategies have been studied. Cevizci *et al*. tested the possibility of analogues of N-acyl homoserine lactone (AHL), a signaling molecule known to play a role in QS systems, to prevent pneumococcal biofilm formation in an *in vivo* cochlear implant model. While these results are preliminary, prolonged treatment with the AHL analogue benzoxazolone after cochlear implant surgery prevented biofilm formation on these implants (Cevizci *et al*. 2015). However, it should be noted that AHL is a molecule solely attributed to Gram-negative bacteria, leaving the exact mechanism of these inhibitors against pneumococci unknown. More recently, a linear molecularly imprinted polymer (LMIP) targeting the TprA receptor and its signaling peptide PhrA was evaluated. Addition of LMIP-PhrA to *in vitro* cultures reduced growth rate and neuraminidase activity, which is important in pneumococcal colonization and invasiveness. In a pneumonia mouse model, dissemination from lungs to blood was prevented by LMIP-PhrA, resulting in longer survival of animals (Motib *et al*. 2017, 2019). Lastly, Yadav *et al*. studied the effect of interfering with DAM or SAM. In 2012, the effect of 5-azacytidine (5-aza) on *in vitro* planktonic and biofilm growth and the effect on gene expression was assessed. 5-aza is a hypomethylating drug used in leukemia treatment. Only a minor effect on planktonic growth was observed, while biofilm formation was adequately and dose-dependently inhibited *in vitro*. Using scanning electron microscopy (SEM), 5-aza-treated biofilms were visualized and observed to be thinner, more scattered in clumps and disorganized compared to non-treated biofilms. Also genes involved in AI-2 synthesis were downregulated after 5-aza treatment (Yadav, Chae and Song 2012). In a very similar study, sinefungin, a natural nucleoside and analogue of SAM known for its inhibitory effects on transmethylation reactions and overall antifungal, antiviral and antiprotozoal activities, was evaluated. Also in this study, *in vitro* biofilm formation was inhibited, and AI-2 synthesis genes were downregulated. Furthermore, *in vivo* biofilm formation in an OM rat model showed a decrease in CFU/bulla after sinefungin treatment (Yadav *et al*. 2014). Inhibition of DAM by the small molecule pyrimidinedione proved equally successful in inhibiting *in vitro* biofilm formation and downregulating virulence-related genes, such as *ply* and *lytB*, as well as the competence-related gene *comC*(Yadav *et al*. 2015).

### Development of novel antibiotics

**Figure 3.**
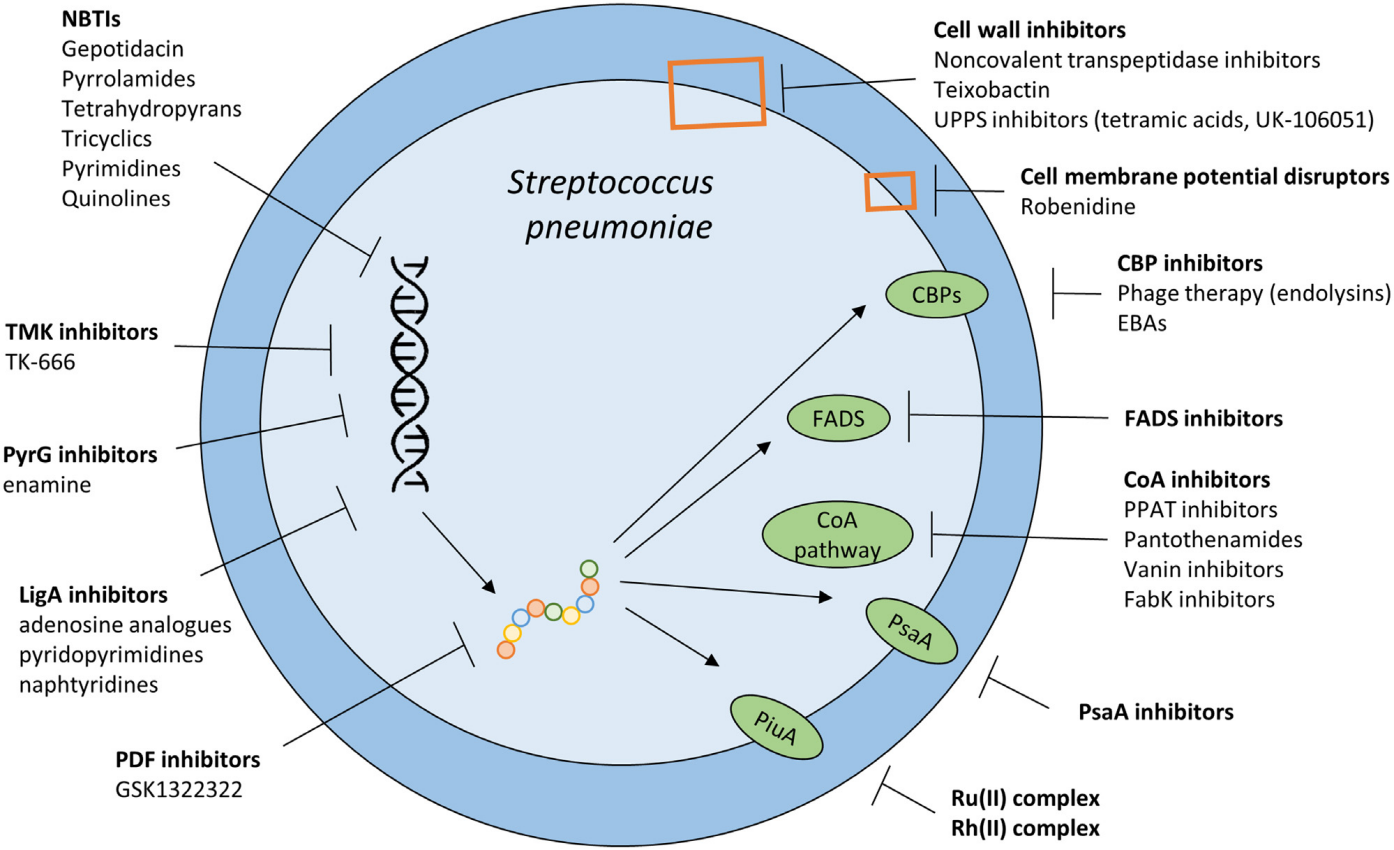
Novel therapies interfering with pneumococcal survival. These therapies often focus on the inhibition of transcription, translation and enzyme elongation. Other strategies include inhibition of cell wall, CBP, FADS, the CoA pathway, PsaA and PiuA. NBTIs: novel bacterial topoisomerase II inhibitors, TMK: thymidylate kinase, LigA: NAD^+^-dependent DNA ligase, PDF: peptide deformylase, UPPS: undecaprenyl pyrophosphate synthetase, CBP: choline binding protein, EBAs: esters of bicyclic amines, FADS: flavin adenine dinucleotide synthetases, CoA: coenzyme A, PPAT: phosphopantetheine adenylyltransferase, PsaA: pneumococcal surface antigen A, Ru: ruthenium, Rh: rhodium.

Lastly, a variety of novel antibiotics is being investigated, which in most cases work against a wider spectrum of pathogens. However, as for in-use antibiotics, mutations leading to resistance are of concern. An overview of the antibiotics tested against pneumococci is shown in Fig. 3. Detailed information regarding the described *in vivo* experiments is listed in Table 6.

**Table 6:** *In vivo* results of novel antibiotics. p.i.: post-infection, CSF: cerebrospinal fluid.

Compound	*In vivo* model	Treatment schedule	Endpoint	References
**Pyrrolamides**	Murine pneumonia model	320 mg/kg orally, starting 18 h p.i., repeated twice per day	4-log reduction in lung burden 42 h p.i.	(Eakin *et al*. 2012)
**Quinolines**	Murine intranasal infection model	50 mg/kg subcutaneously, starting 1 day p.i., repeated twice per day	4-log reduction in lung burden 48 h p.i.	(Odagiri *et al*. 2013)
**Quinolines**	Murine intranasal infection model	40 mg/kg subcutaneously, starting 2h p.i., repeated twice per day	4-log reduction in lung burden 56 h p.i.	(Odagiri *et al*. 2018)
**Tetrahydropyrans**	Murine thigh infection model	80 mg/kg subcutaneously, starting 2 h p.i., repeated every 3 hours for 24 h	4-log reduction in thigh burden 26 h p.i.	(Lepak *et al*. 2016)
**Tricyclics**	Rat pneumonia model	100 mg/kg orally, 1 h, 7 h, 24 h and 31 h p.i.	At least 4-log reduction in lung burden 48 h p.i.	(Miles *et al*. 2016)
**Pyrimidines**	Murine pneumonia model	100 mg/kg intraperitoneally, starting 2 h p.i., repeated 4 times per day	4-log reduction in lung burden 26 h p.i.	(Uria-Nickelsen *et al*. 2013)
**Substituted adenosine analogues**	Murine pneumonia model	45 mg/kg intraperitoneally, starting 18 h p.i., repeated four times per day	5-log reduction in lung burden 36 h p.i.	(Mills *et al*. 2011)
**Teixobactin**	Murine intranasal infection model	10 mg/kg intraveneously, 24 h and 36 h p.i.	6-log reduction in lung burden 48 h p.i.	(Ling *et al*. 2015)
**Endolysins in phages**	Rat meningitis model	20 mg/kg intracisternally or 200 mg/kg intraperitoneally, 18 h p.i.	Rapid decrease in CSF burden after intracisternal (3-log reduction after 30 min) and after intraperitoneal injection (2-log reduction after 3 h)	(Grandgirard *et al*. 2008)
**Endolysins in phages**	Lethal murine sepsis model	25 µg/mouse intraperitoneally, 1 h p.i.	Increase in murine survival from 0% to 70% 7 days p.i.	(Diez-Martinez *et al*. 2015)
**Combination of phages**	Adult zebrafish infection model	3.25 mg/kg total enzyme intraperitoneally, 1 h p.i.	Increase in murine survival from 27.8% to 77.8% 3 days p.i.	(Vázquez and García 2019)
**EBAs**	Embryo zebrafish model	2 µM, starting 7 h p.i., repeated once daily for 3 days	Increase in murine survival from 50% to 97.9% 5 days p.i.	(De Gracia Retamosa *et al*. 2015)
**PPAT inhibitors**	Murine pneumonia model	100 mg/kg intraperitoneally, starting 2 h p.i., repeated twice or 4 times per day	Statis of bacterial burden 24 h p.i.	(De Jonge *et al*. 2013)
**NCL195**	Murine sepsis model	50 mg/kg intraperitoneally, starting 8 h p.i., repeated after 4 h	1-log reduction in burden 18 h p.i., prolonged 60% survival from approx. 26 h p.i. to 36 h p.i.	(Pi *et al*. 2020)

### Bacterial topoisomerase II inhibitors

Bacterial topoisomerases (DNA gyrase and topoisomerase IV) are targets of the well-known quinolones. However, a new class of topoisomerase II inhibitors, called novel bacterial topoisomerase II inhibitors (NBTIs), has been put forward as a new way of treating quinolone-resistant infections. These inhibitors also bind to DNA gyrase and topoisomerase IV, yet do so on a slightly different binding site, thereby evading existing resistance mechanisms. Gepotidacin represent this new drug class. It shows *in vitro* activity against a variety of Gram-positive bacteria, including quinolone-resistant pneumococci (Bax *et al*. 2010; Biedenbach *et al*. 2016; Flamm *et al*. 2017). Gepotidacin is currently recruiting for phase III trials, evaluating efficacy, safety and applicability of the drug (Kolarič, Anderluh and Minovski 2020; Koulenti *et al*. 2020). Other classes of bacterial topoisomerase II inhibitors are the pyrrolamides, tetrahydropyrans, tricyclics, pyrimidines and quinolines. For some of these lead compounds, only *in vitro* efficacy has been tested, while others also show promising effects *in vivo*. Regardless, none of these classes have progressed into clinical trials for pneumococcal infections (Zhang *et al*. 2011b; Eakin *et al*. 2012; Mitton-Fry *et al*. 2013; Odagiri *et al*. 2013, 2018; Uria-Nickelsen *et al*. 2013; Surivet *et al*. 2015; Lepak *et al*. 2016; Miles *et al*. 2016).

### Thymidylate kinase (TMK) inhibitors

TMK is an essential enzyme in the DNA synthesis pathway. It transfers phosphate from adenosine triphosphate (ATP) to thymidine monophosphate (dTMP), which leads to the formation of thymidine diphosphate (dTDP), an essential component of the thymidine triphosphate (dTTP) pathway (Keating *et al*. 2012). As such, TMK is essential for bacterial growth and has been put forward as an interesting drug target (Petit and Koretke 2002). Structure-based drug design led to the development of several compounds, of which TK-666 has proven to be active *in vitro* against Gram-positive bacteria, while not being harmful to eukaryotic cells. Furthermore, it was shown to be equally active against antibiotic resistant and susceptible pneumococci (Keating *et al*. 2012; Martínez-Botella *et al*. 2012). Currently, TMK inhibitors are mainly evaluated for their use against *Mycobacterium tuberculosis* infections (Jian *et al*. 2019, 2020; Venugopala *et al*. 2020).

### PyrG inhibitors

PyrG, a bacterial cytidine triphosphate (CTP) synthase, produces CTP from uridine triphosphate (UTP) and glutamine and is essential in the pyrimidine *de novo* biosynthetic pathway (Endrizzi *et al*. 2004). Furthermore, it is required for growth of bacteria such as *Haemophilus influenzae*. PyrG inhibitors were first reported in the literature over 40 years ago as antitumor drugs, however, thus far no PyrG inhibitors have reached the market. After an enzyme-based HTS of PyrG inhibitors, enamine proved the most interesting. However, while the IC_50_ value in the enzymatic assay was low (0.091 µM), the MIC was more than 128 µg/mL for pneumococci. In contrast, MICs ranging from 16 to 64 µg/mL were observed for other pathogens such as *H. influenzae*, *E. coli* and *S. aureus*. While this demonstrates the necessity of whole-cell based assays in drug discovery, it is also a first step towards the development of pneumococcal PyrG inhibitors (Yoshida *et al*. 2012). Similarly to TMK inhibitors, PyrG inhibitors are now primarily studied as a way to combat mycobacterial infections (Chiarelli *et al*. 2018).

### NAD^+^-dependent DNA ligase (LigA) inhibitors

LigA is an essential enzyme in viability for both Gram-positive and Gram-negative bacteria. It is completely unrelated to eukaryotic DNA ligases, making it an interesting drug target (Pascal 2008; Mills *et al*. 2011). Selective inhibitors of LigA, substituted adenosine analogues, were reported to show a broad spectrum of bacterial inhibition, ranging from *E. coli*, *Mycoplasma pneumoniae* to *S. pneumoniae*. No binding affinity to eukaryotic DNA ligases or cytotoxicity against human red blood cells or alveolar epithelial cells A549 was observed. Furthermore, an *in vivo* murine lung infection model showed a dose-dependent reduction of pneumococci after treatment with one of these adenosine analogues (Mills *et al*. 2011). Optimization of compound classes pyridopyrimidines and naphthyridines has also been studied *in vitro*, leading to LigA inhibiting molecules for a wide range of bacteria, including pneumococci (Murphy-Benenato *et al*. 2015). More recently, the mechanism of action of the long known broad-spectrum antibiotic cordycepin was attributed to its binding to LigA (Zhou *et al*. 2016). However, no follow-up research on any of the aforementioned compounds has been performed.

### Peptide deformylase (PDF) inhibitors

PDF is a metalloenzyme used in bacterial peptide elongation. It cleaves a formyl group from the terminal N-methionine of a newly synthesized polypeptide following ribosomal translation and elongation. Removal of this formyl group is vital for bacterial viability. Importantly, human PDF is structurally different from its prokaryotic counterpart, therefore bacterial PDF is considered an interesting novel drug target (Sangshetti, Khan and Shinde 2014). In 2011, GSK1322322 was introduced as a potential novel antibiotic against multidrug resistant *S. aureus* and CABP (Ross *et al*. 2011). It showed good *in vitro* activity against pneumococci and was able to reduce pneumococcal lung burdens in a murine model (Sutcliffe 2011). This molecule was undergoing clinical trials, however trials were ended due to the identification of potentially harmful metabolites.(United States National Institute of Health (NIH) 2019) IDP-73 152, another PDF inhibitor, went through a phase I clinical study in 2013, showing no adverse effects after oral administration in healthy volunteers. The results were published in 2019, but no follow-up studies have been announced (Shin *et al*. 2019).

### Inhibition of cell wall synthesis

Beta-lactams, the most widely used antibiotics, interfere with cell wall synthesis by targeting the transpeptidase activity of penicillin-binding proteins (PBPs). They block transpeptidation by covalently binding to these enzymes, thereby killing the bacteria. However, resistance is increasing for this antibiotic class.(Macheboeuf *et al*. 2006) Therefore, attempts have been made to discover novel inhibitors. One of these strategies led to the screening of noncovalent inhibitors, as they are postulated to be less susceptible to resistance-inducing mutations of PBPs and production of beta-lactamases (Turk *et al*. 2011). In 2009, the first virtual screening of noncovalent pneumococcal inhibitors was performed. However, no biological assays were performed to confirm these results (Miguet *et al*. 2009). Another article from 2011 reported on 2 molecules showing promising *in vitro* activity against antibiotic-resistant pneumococci (Turk *et al*. 2011). Since then, no results on noncovalent PBP inhibitors have been published, suggesting this approach was unsuccessful.

Using a completely different mechanism, Lewis *et al*. published the finding of the first novel natural bacterium-derived antibiotic in decades (Durand, Raoult and Dubourg 2019). Teixobactin inhibits peptidoglycan biosynthesis by binding to lipid II and lipid III, precursors of peptidoglycan and teichoic acid, respectively. *In vitro*, it is active against a variety of Gram-positive bacteria, but not against Gram-negatives. Teixobactin showed a dose-dependent reduction in pneumococcal lung burden in a murine model (Ling *et al*. 2015). Since its discovery, numerous analogues have been synthesized, however still no clinical trials involving teixobactin or one of its analogues have been setup (McCarthy 2019; Koulenti *et al*. 2020).

Lastly, undecaprenyl pyrophosphate synthetase (UPPS) synthesizes undecaprenyl pyrophosphate (UPP) from isopentenyl pyrophosphate (IPP). UPP is essential in the bacterial cell wall synthesis, as it is needed for cross membrane transport of carbohydrates, and as such, UPPS has been shown to be essential for pneumococcal growth (Apfel *et al*. 1999). However, only few attempts have been made to develop UPPS inhibitors against pneumococci. In 2010, tetramic acids were evaluated as pneumococcal UPPS inhibitors. While these compounds showed interesting *in vitro* activity, no whole-cell based assays were performed (Lee *et al*. 2010). Later, Danley *et al*. reported *in vitro* UPPs inhibition by UK-106 051, a carboxamide analogue, leading to inhibition of pneumococcal growth, yet no *in vivo* confirmation has been attempted (Danley *et al*. 2015). Since then, few papers on the use of UPPS inhibitors against a variety of bacteria were published, all reporting early stage preclinical data (Concha *et al*. 2016; Inokoshi *et al*. 2016; Wang *et al*. 2016; Cherian *et al*. 2017; Jukic *et al*. 2019). While these data show potential for this target, currently there are no UPPS inhibitors in development.

### Choline binding protein (CBP) interference

CBPs are a large group of proteins located on the pneumococcal cell surface, involved in pneumococcal autolysis (LytA, LytC, CbpD, CbpF), cell separation after division (LytB), interference with complement activation (PspA), adherence to host cells (PspC, PcpA, CbpG) and modulating the amount of choline (Pce). There are two main strategies to use CBP as a novel anti-pneumococcal approach, (i) through direct inhibition of CBPs and (ii) through the administration of endolysins (Maestro and Sanz 2016).

Maestro *et al*. published a detailed review in 2016 on the use of direct CPB inhibitors (Maestro and Sanz 2016). All inhibitors were based upon choline, such as esters of bicyclic amines (EBAs) which can be used as monomers or nanoparticle dendrimers, consisting of several ligands. As CBPs contain several binding sites, the binding affinity increases dramatically (Mammen, Choi and Whitesides 1998). De Gracia Retamosa *et al*. showed EBAs were capable of lysing *in vitro* planktonic cultures in a dose-dependent manner and increased survival in a zebrafish model (De Gracia Retamosa *et al*. 2015). In a 2019 study, EBAs were confirmed to *in vitro* lyse planktonic cultures using a LytA-dependent mechanism. Furthermore, also *in vitro* formation of biofilms was blocked (Roig-Molina *et al*. 2019). Lastly, phagocytosis of bacteria by microglial cells increased in *in vitro* dendrimer-treated pneumococcal cultures (Ribes *et al*. 2013).

Phage therapy has repeatedly been suggested in the battle against multidrug resistant bacteria. Bacteriophages have been observed to kill antibiotic resistant bacteria. In addition, some of their products, such as endolysins, show promising results (Cisek *et al*. 2017). Interestingly, phage resistance mostly comprises a loss of bacterial virulence (Caflisch, Suh and Patel 2019). Endolysins are produced by phage-infected bacteria to enable the release of novel phages, thereby destroying the bacterial cell wall.(Stoffels *et al*. 2017) In pneumococci—and other Gram-positive bacteria—these lysins belong to the CBP family. Therefore, they recognize choline residues in the teichoic acids of pneumococci (Vázquez and García 2019). Administration of purified endolysins to *in vitro* pneumococcal cultures led to a rapid decrease in cell density (Diez-Martinez *et al*. 2015). Furthermore, mice and rats showed a greater likelihood of survival after endolysin therapy following a pneumococcal challenge (Grandgirard *et al*. 2008; Diez-Martinez *et al*. 2015). Synergy between different phages has been observed both *in vitro* on planktonic and biofilm cultures and in a zebrafish model, leading to an increase in zebrafish survival (Vázquez and García 2019). Several clinical trials involving phage therapy have already been done, or are ongoing or planned (Vázquez, García and García 2018; Caflisch, Suh and Patel 2019). While none of these trials have focused on pneumococci, this does indicate phages or lysins are promising antibacterial drug candidates.

### PsaA inhibitors

Apart from enhancing the immune system through passive immunization (see earlier), PsaA has also been proposed as a direct drug target. Inhibitors have been studied for their anti-pneumococcal properties. In 2015, Bajaj *et al*. virtually screened a library of small molecules for their binding properties to PsaA (Couñago *et al*. 2014). Hits were further optimized and tested in an *in vitro* model. Two molecules showed promising inhibitory properties against pneumococci, however the authors stated that more optimization of these compounds was needed (Bajaj *et al*. 2015). This experimental setup was later repeated by another research group, confirming PsaA as an alternative drug target. However, this research group also failed to identify a sufficiently inhibiting molecule, as its most active compounds possessed a flexible tail, which is considered a poor development prospect (Obaidullah *et al*. 2018).

### Flavin adenine dinucleotide synthetase (FADS) inhibitors

FADSs synthesize flavin mononucleotide (FMN) and flavin adenine dinucleotide (FAD). As cofactors of flavoproteins, FMN and FAD are present in all living organisms and insufficiency of either leads to cell death. In bacteria, FADS synthesizes FMN and FAD from riboflavin (RF) in two steps: production of FMN from RF (RFK module) and subsequent production of FAD from FMN (FMNAT module). While there is some similarity between pro- and eukaryotes in the RFK module, this is not the case for the FMNAT module, making it an interesting novel drug target (Serrano *et al*. 2013; Sebastián *et al*. 2017). Using an enzyme-based HTS approach based on the crystal structure of *Corynebacterium ammoniagenes*, several lead compounds were identified. However, biological activity against pneumococci remained low, suggesting they do not reach inhibitory concentrations at the intracellular level (Sebastián *et al*. 2018). Lastly, a recent study using virtual screening identified four FADS inhibitors inhibiting pneumococcal growth *in vitro* (Lans *et al*. 2020).

### Coenzyme A (CoA) pathway inhibitors

CoA is an essential cofactor in all living organisms in the metabolism of fatty acids. It is synthesized from pantothenate (vitamin B5), cysteine and ATP. First, pantothenate is phosphorylated to 4’-phosphopantothenate by pantothenate kinase (CoaA). This molecule is then condensated with cysteine and subsequently decarboxylated to obtain 4’-phosphopantetheine. Using the enzyme phosphopantetheine adenylyltransferase (PPAT) and ATP, 4’-phosphopantetheine is converted to dephospho-CoA, which is subsequently phosphorylated to yield CoA (Leonardi *et al*. 2005).

PPAT is an essential enzyme within the CoA pathway. It has been put forward as a novel drug target numerous times, because of its structurally attractive site, high degree of conservation among bacterial species, distinct differences between bacterial and human PPAT, known kinetics and available purified enzyme (Miller *et al*. 2010). In 2013, de Jonge *et al*. identified several lead compounds capable of inhibiting PPAT through structure-based HTS. Moreover, these compounds inhibited *in vitro* growth of macrolide-resistant pneumococci, as well as other antibiotic-resistant Gram-positive bacteria. In a murine lung infection model, treatment with PPAT inhibitors led to a stasis of bacterial burden (De Jonge *et al*. 2013).

Furthermore, pantothenamides, derivates of pantothenate (vitamin B5), are known to possess antibiotic activity *in vitro*. They are substrates of CoaA, leading to the formation of inactive CoA analogues (Zhang, White and Rock 2006). In mammals, vanins—also called panthetheinases—hydrolyze pantetheine into vitamin B5, as a way of vitamin recycling (Jansen *et al*. 2013a). In 2013, studies showed that antibiotic pantothenamides were also hydrolyzed and thus inactivated by vanins, leading to the combination of pantothenamides and vanin inhibitors as a novel antibacterial strategy against Gram-positive bacteria. Vanin inhibitors were able to protect pantothenamides from degradation by host panthetheinases, making pantothenamides more stable for use (Jansen *et al*. 2013b). Furthermore, pantothenamides resistant towards vanin activity have been reported recently. However, activity of these modified pantothenamides towards pneumococci has not been assessed yet, as they are now mainly studied for their antiplasmodial activity (Jansen *et al*. 2019; Spry *et al*. 2020).

In turn, CoA is used in a series of reactions to produce fatty acids. In these subsequent reactions, enoyl-acyl carrier protein reductase (ENR) catalyzes the last and rate-limiting step in each round of chain elongation. Different isoforms of ENR exist, however, pneumococci only possess one of them, called FabK (Heath and Rock 1995). *In silico* docking revealed several potential inhibitors for FabK, however, to date, none of these have been tested further (Zhang *et al*. 2011a). Some ENR inhibitors have reached clinical trials, however, none of them are specific to FabK (Rana *et al*. 2020).

### Interference with iron transport systems

Iron is an essential nutrient for bacterial growth and survival. As the concentration of free iron in the host is low, bacteria developed highly specific iron-acquisition systems on their membrane surfaces. In *S. pneumoniae*, PiaABC, PiuABC and PitABC are the three known iron-transport systems, respectively responsible for the acquisition of heme, ferrichrome and ferric irons (Brown *et al*. 2002; Cheng *et al*. 2013). PiaA, PiuA and PitA located on the cell surface bind these free iron-molecules. A Ru(II) complex has been tested for its inhibitory activity towards PiuA, needed for ferrichrome transport. This complex was capable of inhibiting pneumococcal growth without affecting an alveolar epithelial cell line *in vitro* (Yang *et al*. 2014a). A similar study conducted with a Rh(II) complex showed the same effects, consolidating the use of metal complexes as potential novel anti-pneumococcal drugs (Yang *et al*. 2019a). Since metal complexes only recently drew attention as potential antibacterial agents, development is still in a very early stage (Frei 2020).

### Repurposing of existing drugs

Repurposing of existing drugs has important benefits. As these drugs are already in use, their safety, tolerability and toxicity has been extensively studied, reducing the costs for development (Cragg, Grothaus and Newman 2014).

Robenidine is an anticoccidial agent used worldwide in poultry and rabbits. Recently, it has been evaluated for its activity on bacteria such as pneumococci and *S. aureus*. Robenidine, together with two analogues, showed *in vitro* bactericidal activity against pneumococci by disrupting the cell membrane potential, leading to a thicker cell membrane and a wider periplasmic space (Ogunniyi *et al*. 2017). Recently, treatment of septic mice with one of these analogues, NCL195 showed a minor reduction in bacterial burden 18 h p.i. Survival was also prolonged, as all mice died 16 h p.i. in the control group. In the treatment group, 60% was still alive at this point, but eventually all mice succumbed at 46 h p.i. As such, NCL195 is not suitable for further development, but its scaffold could be used in further research (Pi *et al*. 2020).

Choline kinase (ChoK) is a mediator of cell growth and division of eukaryotic cells. As such, it is a drug target for tumor cells. ChoK inhibitor RSM-932A is undergoing clinical trials. However, multiple bacteria including pneumococci also express ChoK. Human ChoK inhibitors MN58b and RSM-932A have been shown to inhibit pneumococcal growth *in vitro*. However, as these inhibitors also inhibit human ChoK and potentially other bacterial ChoK, including those of the human microbiome, other inhibitors need to be identified (Zimmerman, Lacal and Ibrahim 2019).

Antioxidants N-acetyl-L-cysteine and cysteamine are mucolytics and have been proposed suitable in the treatment of Huntington's and Parkinson's diseases, as well as cystic fibrosis and malaria. *In vitro*, these two compounds have antibacterial activity against pneumococci in mixed-species biofilms with *H. influenzae*, killing 98% of all bacteria (Domenech and García 2017). However, it should be noted pneumococci used in this study were non-encapsulated to promote biofilm formation, which might affect results.

Lastly, auranofin, a compound used in the treatment of rheumatoid arthritis, has been tested *in vitro* against multiple bacteria including multidrug resistant pneumococci. Auranofin and derivative MH05 were subsequently tested in a murine sepsis model. They were shown to significantly reduce mortality and bacterial burden of infections with several pneumococcal strains (Aguinagalde *et al*. 2015). Currently, auranofin is in phase 2 clinical trials as therapy against *M. tuberculosis*(Butler and Paterson 2020).

## CONCLUSION

Mostly in academia, major efforts are underway to identify potential new drug targets and to improve infection outcomes. Currently, there are three main strategies: (i) boosting host immunity by interfering with its immune responses, (ii) lowering pneumococcal virulence and (iii) developing novel antibacterials with a new mechanism of action (MOA). Each of these strategies has its own benefits and limitations. The first strategy often leads to adjuvant therapies, which can be used in concurrence with current antibiotics. While some of these therapies show highly promising results, no clinical trials have been set up to date. Importantly, interfering with host immune responses is a very delicate process. The biggest limitation to developing therapies interfering with host immunity is the risk of creating immune imbalances which can pose serious health threats (Bewersdorf *et al*. 2018). Still, this strategy is gaining attention, as publications increase and clinical trials are initiated. Secondly, lowering pneumococcal virulence can be done in multiple ways. The general idea is to inhibit important virulence factors to enable our own immune system to overcome the infection, without the need for bactericidal compounds. However, most of the proposed drug targets are pathogen specific. Notwithstanding their beneficial effects on disease outcome, use of these inhibitors requires certainty regarding the causative agent, which cannot always be guaranteed. Lastly, the development of novel natural antibacterials is hampered by the difficult culturing methods of soil bacteria, the primary source for natural antibiotics. Followed by the discovery of teixobactin, this issue was partially resolved by the development of the iChip technology (Ng and Chan 2016). Furthermore, while rational drug discovery (e.g. based on enzymatic screenings) shows promising results, the pharmaceutical industry is not keen on investing in novel antibiotics as investment costs are high, while the profits could be small (Arias and Murray 2015; Jackson, Czaplewski and Piddock 2018). Even when a potential hit is successfully identified, the emergence of resistance is always on the lure, lowering return on investments (Wright 2015). Therefore, the Infectious Diseases Society of America (IDSA) presented the 10 x ’20 initiative ten years ago, in 2010, to develop and approve 10 novel, efficacious and safe systemically administered antibiotics by 2020 (Gilbert *et al*. 2010). While the initial goal is met, in 2019 IDSA released a publication stating that even the development of 20 novel antibiotics might still not be sufficient to tackle future drug resistance problems (Talbot *et al*. 2019). Therapies targeting the bacteria without killing them, i.e. anti-virulence therapies, might provide an answer to this issue. However, most research involving these targets is still in an early stage, with only liposomes as PLY inhibitors currently going through clinical trials.

## MATERIALS AND METHODS

### Pubmed searches

‘Streptococcus pneumoniae pipeline’, ‘streptococcus pneumoniae novel drug target’, ‘streptococcus pneumoniae novel drug’, ‘streptococcus pneumoniae novel therapy’, ‘(‘Therapeutics’[Mesh] OR ‘Anti-Infective Agents’[Mesh]) AND (‘Streptococcus pneumoniae’[Mesh] OR ‘Pneumococcal Infections’[Mesh]) AND novel’. The literature reporting on anti-pneumococcal activity without explicit bacterial target (e.g. evaluation of plant extracts) was excluded. The literature reporting on novel drug targets older than 10 years (cut-off January 2010) was excluded.
